# Phosphoribosyl modification of poly-ubiquitin chains at the *Legionella*-containing vacuole prohibiting autophagy adaptor recognition

**DOI:** 10.21203/rs.3.rs-3266941/v1

**Published:** 2023-09-14

**Authors:** Min Wan, Marena E. Minelli, Qiuye Zhao, Shannon Marshall, Haiyuan Yu, Marcus Smolka, Yuxin Mao

**Affiliations:** a Weill Institute for Cell and Molecular Biology, Cornell University, Ithaca, NY 14853, USA.; b Department of Molecular Biology and Genetics, Cornell University, Ithaca, NY 14853, USA.; c Department of Computational Biology, Cornell University, Ithaca, NY 14853, USA.

## Abstract

Ubiquitination is a crucial posttranslational modification in eukaryotes that plays a significant role in the infection of intracellular microbial pathogens, such as *Legionella pneumophila,* the bacterium responsible for Legionnaires’ disease. While the *Legionella*-containing vacuole (LCV) is coated with ubiquitin (Ub), it avoids recognition by autophagy adaptors. In this study, we report that the Sdc and Sde families of effectors work together to build ubiquitinated species around the LCV. The Sdc effectors catalyze canonical polyubiquitination directly on host targets or on the phosphoribosyl-Ub (PR-Ub) conjugated to host targets by Sde. Remarkably, the Ub moieties within the poly-Ub chains are either modified with a phosphoribosyl group by Sde and other PDE domain-containing effectors or covalently attached to other host substrates via Sde-mediated PR-ubiquitination. Furthermore, these modifications prevent the recognition by Ub adaptors, such as p62, and therefore exclude host autophagy adaptors from the LCV. Our findings shed light on the nature of the poly-ubiquitinated species present at the surface of the LCV and provide a molecular mechanism for the avoidance of autophagy adaptors by the Ub-decorated LCV.

## Introduction

Autophagy is a major degradation pathway that is activated in various stress conditions such as starvation, organelle damage, and pathogen invasion ^1, 2^. Autophagy is initiated by the activation of the ULK1 serine/threonine kinase, which assembles a complex with FIP200, ATG13, and ATG101 at the phagophore formation site ^3^. Activated ULK1 promotes the recruitment of class III PI3K complex to the phagophore, where PI(3)P is generated to function in autophagosome nucleation ^4^. The expansion and maturation of autophagosomes are facilitated by two ubiquitin-like conjugation systems, through which, the ubiquitin-like LC3 is conjugated to phosphatidylethanolamine (PE) at both the inner and outer membranes of the phagosome ^5, 6^. Mature phagosomes need to fuse with lysosomes to achieve autophagic degradation. Although autophagy was originally discovered as a nonselective bulk degradation process in response to starvation ^7^, selective autophagy that targets specific substrates for degradation was later characterized by myriad studies ^8^. Many types of selective autophagy involve the ubiquitination of cargoes, which are then recognized by ubiquitin-binding autophagy adaptor proteins (autophagy receptors). Specific autophagy adaptors, such as p62/SQSTM1, NDP52, and OPTN contain ubiquitin-binding domains and conserved LC3-interacting regions (LIR) and recruit ubiquitinated cargoes to the LC3-positive autophagy compartment ^9^.

Xenophagy, one type of ubiquitin-dependent selective autophagy, is a key mechanism for the host to defend against the invasion of intracellular pathogens, such as *Shigella flexneri, Listeria monocytogenes*, and *Salmonella enterica* ^10, 11^. During infection, Ub can be attached to pathogens or pathogen-containing vacuoles by ubiquitin E3 ligases to generate a “Ub-coat” surrounding the pathogen. The “Ub-coat” serves as a signaling platform to recruit autophagy adaptors and thus drive the sorting of the cargo into the autophagic vacuole for degradation. Among the autophagy adaptors, p62 is the most well-studied molecule that is involved in the xenophagy of many kinds of pathogens ^8^.

*Legionella pneumophila* is an opportunistic intracellular bacterial pathogen that causes a severe form of pneumonia called Legionnaires’ disease ^12, 13^. After *L. pneumophila* is phagocytosed in host cells, this bacterium secrets over 300 effector proteins into the host through the Dot/Icm, a type IV secretion system (T4SS) ^14, 15^. These bacterial effector proteins hijack various host cellular pathways to create an optimal niche, the *Legionella*-containing vacuole (LCV) for robust bacterial intracellular growth ^16^. Among these host cellular pathways, the ubiquitination pathway is a prime target of this intracellular pathogen. Ubiquitination is one of the most essential eukaryotic posttranslational modifications (PTMs) that regulates a plethora of physiological processes, including protein homeostasis ^17^, cell signaling ^18^, and membrane trafficking ^19, 20^. It has been shown that the surface of the LCV is positive for Ub signals as early as 1 hour after infection and remains positive throughout the infection ^21^. Later studies revealed that the “Ub-coat” around the LCV contains both K48 and K63-linked Ub chains ^22^. The *Legionella* Sdc family of effectors (SidC and SdcA), which have a unique HECT-type Ub E3 ligase domain, have been shown at least partially to be responsible for the accumulation of ubiquitinated species around the LCV ^23, 24^. Besides the Sdc effectors that catalyze canonical ubiquitination, the Sde family of effectors (SidE, SdeA, SdeB, and SdeC) also play a role in the accumulation of ubiquitin signals at the LCV ^25^. Sde effectors are a group of novel Ub ligases that act independently of ATP, Mg^2+,^ or E1 and E2 enzymes ^26–28^. The Sde family enzymes first catalyze ADP-ribosylation on Ub to generate mono-ADP-ribosyl Ub (ADPR-Ub), via the mono-ADP-ribosyl transferase (mART) domain. Next, the intermediate product, ADPR-Ub can be either conjugated to serine or tyrosine residues on substrates (phosphoribosyl- or PR-ubiquitination) or hydrolyzed to phosphoribosyl-Ub (PR-Ub) via its phosphodiesterase (PDE) domain ^29–35^.

Surprisingly, although the LCV is enriched with ubiquitinated species, ubiquitin-binding autophagy adaptors are excluded from the LCV ^36^. Previous studies showed that *L.pneumophila* applies multiple strategies to avoid host autophagy. The *Legionella* effector RavZ irreversibly deconjugates lipidated LC3 on autophagosomal membranes and thus prevents autophagy-mediated degradation of the LCV ^37^. Another effector LpSPL hydrolyzes host sphingolipids and inhibits basal autophagy ^38, 39^. Furthermore, The *Legionella* effector Lpg1137 was identified as a serine protease responsible for the cleavage of syntaxin 17 and hence blocks autophagy and BAX-induced apoptosis ^40, 41^. These findings suggest that multiple mechanisms may exist for the bacterium to avoid clearance by autophagy.

In this study, we demonstrate the cross-talk between the Sdc and Sde families of effectors. We show that Sdc and Sde work together to build mixed poly-Ub chains on host substrates and both of these two families of effectors are required for the accumulation of ubiquitinated species around the LCV. Surprisingly, Sde proteins, further modify the poly-Ub chain by phosphoribosylation on residue Arg42 of Ub moieties or directly conjugate the poly-Ub chain to other host substrates via PR-ubiquitination. These modifications block the binding by the autophagy adaptor p62 to the Ub-enriched LCV and serves as an alternative mechanism to evade host autophagy.

## Results

### Both the Sdc and Sde effectors are required for the enrichment of ubiquitinated species at the LCV

**Figure 1.**
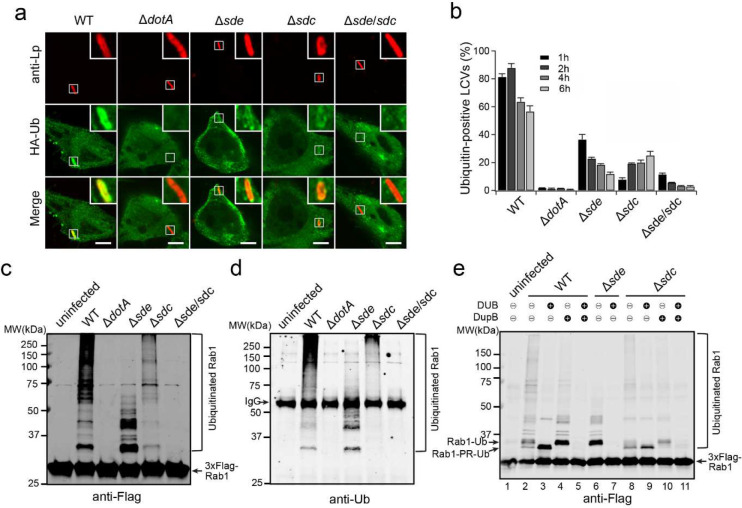
The *sde* and *sdc* families of effectors are required for the ubiquitination of host targets and the enrichment of ubiquitinated species at the LCV. (a) Representative confocal images showing the Ub signals (green) at the LCV in HEK293T cells challenged with specified *Legionella* strains (red) at 1 hpi. Scale Bar: 5 μm. (b) Quantitative analysis of Ub-positive LCVs in cells infected with the indicated *Legionella* strains. Data were shown as means ± SEM of three independent experiments. At least 60 randomly selected LCVs were counted for each condition. (c) Western-blot analysis of Rab1 ubiquitination. HEK293T cells were transfected with plasmids expressing FcγRII and 3xFlag-Rab1 and then infected with the indicated *Legionella* strains for 1 hour. Cell lysates were prepared and 3xFlag-Rab1 was immunoprecipitated using anti-Flag resins. Precipitated materials were analyzed by Western blot using anti-Flag or (d) anti-Ub antibodies. (e) Western-blot analysis 3xFlag-Rab1 after immunoprecipitation from cells challenged with indicated *Legionella* strains followed by DUB and/or DupB treatment.

Previous studies reported that shortly after *L. pneumophila* infection, ubiquitinated conjugates were accumulated at the bacterial phagosome ^21, 22^. The Sdc enzymes, which function as HECT-type E3 ligases, were shown to be required for the enrichment of Ub signals at the LCV ^23, 42^. Interestingly, the Sde proteins were also shown to influence Ub signals at the LCV ^25^. To elucidate the roles of these two distinct families of Ub ligases in LCV biogenesis, we analyzed the dynamics of Ub signals at the LCV during *L. pneumophila* infection. FcγRII-expressing HEK293T cells were challenged with the wild-type (WT) *L. pneumophila* Lp02 strain, approximately 80% of LCVs were stained positive for Ub at 1 hour post infection (hpi) and then gradually decreased at later time points of infection (Fig. 1a, b). The accumulation of ubiquitinated species around the LCV was dependent on the Dot/Icm secretion system as no Ub signals were detected in cells infected with the Δ*dotA* mutant strain. In agreement with previous results ^23, 42^, the enrichment of Ub signals at the LCV was markedly reduced (~8%) in cells challenged with the Δ*sdc* (*sidC* and *sdcA*) strain at 1 hpi. However, the percentage of Ub-positive LCVs recovered, albeit to a less extent compared to the WT strain, at later time points of infection (~19% at 2 hpi and ~25% at 6 hpi) (Fig. 1a, b). The Ub signals at the LCV were also substantially reduced in cells challenged with the Δ*sde* (*sidE*, *sdeA, sdeB, and sdeC*) strain with ~36% of LCVs positive for Ub at 1 hpi. In contrast to the Δ*sdc* strain, the percentage of Ub-positive LCVs further dropped at later infection time points (as low as ~12% at 6 hpi) (Fig. 1a, b) suggesting Sde effectors are important in stabilizing ubiquitinated species at the LCV. Strikingly, the Ub signals were nearly completely abolished at the LCV in cells infected with the Δ*sde/sdc* strain (Fig. 1a, b). These observations suggest that both Sdc and Sde are the primary factors responsible for the enrichment of ubiquitinated species at the LCV.

### Multiple host proteins are modified by both Sdc and Sde during *Legionella* infection

**Extended Data Figure 1.**
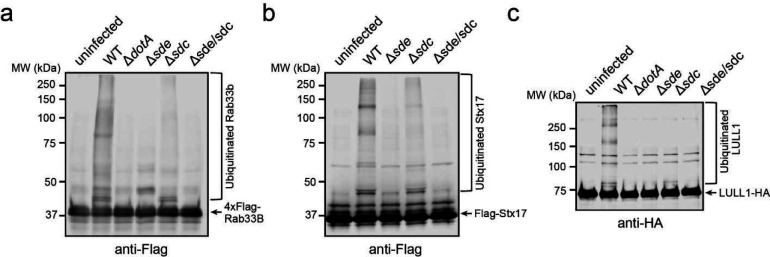
Rab33B, Stx17, and LULL1 ubiquitination by the Sdc and Sde families of effectors during *Legionella* infection. HEK293T cells were transfected with 4xFlag-Rab33B (a), Flag-Stx17 (b), or LULL1-HA (c) and then infected with relevant *Legionella* strains for 1 hr. Rab33B, Stx17, and LULL1 were enriched by anti-Flag or anti-HA immunoprecipitation and analyzed by anti-Flag or anti-HA Western blot.

It has been shown that some host proteins, such as Rab1 and Rab10, are targeted by both Sdc and Sde families of effectors ^23, 26, 43^. We hypothesized that the accumulation of ubiquitinated species at the LCV is due to direct ubiquitination and PR-ubiquitination on the same host targets by Sdc and Sde, respectively. To test whether Sdc and Sde work together to modify the same host targets, we first analyzed the ubiquitination state of Rab1 in cells after *L. pneumophila* infection. HEK293T cells were transfected with plasmids expressing 3xFlag-Rab1 and FcγRII for 24 hours and were challenged with WT or mutant *Legionella* strains for 1 hour. Rab1 was then immunoprecipitated and analyzed by Western blot (Fig. 1c, d). Strong ubiquitinated Rab1 signals were detected in samples infected with WT *Legionella* strain but the signals were notably reduced in samples infected with either the Δ*sde* or Δ*sdc* strain. Furthermore, the ubiquitinated Rab1 signals were nearly abolished in the samples infected with the Δ*sde*/*sdc* strain. The Sdc and Sde-dependent ubiquitination/PR-ubiquitination of host targets was not limited to Rab1 as several other previously reported Sde substrates, Rab33B ^26^, the autophagosomal SNARE protein Stx17 ^44^, and an ER membrane protein, LULL1 ^45^ showed a similar Sdc and Sde-dependent ubiquitination during *Legionella* infection (Extended data Fig. 1a–c). Our data suggest that multiple host proteins can be both ubiquitinated and PR-ubiquitinated by Sdc and Sde.

**Extended Data Figure 2.**
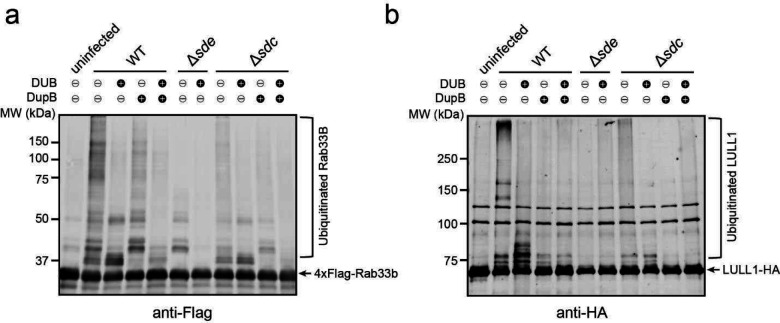
Multiple host substrates were ubiquitinated by Sdc and Sde in Legionella infection. (a) Western-blot analysis 4xFlag-Rab33B after immunoprecipitation from cells challenged with indicated *Legionella* strains followed by DUB and/or DupB treatment. (b) Western-blot analysis LULL1-HA immunoprecipitation from cells challenged with indicated *Legionella* strains followed by DUB and/or DupB treatment.

This observation was further supported by the treatment of immunoprecipitated substrates with a canonical DUB (the N-terminal deubiquitinase domain of SdeA) ^25^ and/or the PR-ubiquitination specific deubiquitinase (DupB) ^45^. Immunoprecipitated Rab1 was first prepared from cells infected with *Legionella* strains and was treated with deubiquitinases before Western blot analysis. The high molecular weight bands, corresponding to the poly-ubiquitinated Rab1, were substantially reduced after the treatment of either DUB or DupB (Fig. 1e, lanes 3 and 4). Strikingly, ubiquitinated Rab1 was completely abolished after the treatment with both enzymes (Fig. 1e, lane 5). Along this line, ubiquitinated Rab1 prepared from cells infected with Δ*sde* or Δ*sdc* strain was also markedly reduced after DUB or DupB treatment (Fig. 1e, lane 6–11). Similarly, the reduction of ubiquitinated forms of other host substrates, such as Rab33B and LULL1 was evident after DUB or DupB treatment (Extended data Fig. 2a, b). Together, these data demonstrated that multiple host proteins can be simultaneously ubiquitinated (via Sdc) and PR-ubiquitinated (via Sde) during *Legionella* infection. It is also worth noting that in samples from cells infected with the Δ*sdc* strain, ubiquitinated Rab1 was still detected even after the DupB treatment (Fig. 1e, lane 8–11), indicating Rab1 may also be ubiquitinated by other canonical E3 ligases (see discussion below).

### Ubiquitination by Sdc and Sde promotes the recruitment of host substrates and ER membranes to the LCV

**Extended Data Figure 3.**
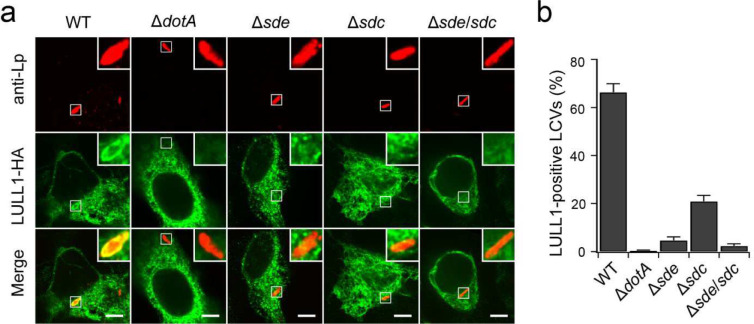
Sde and Sdc facilitate the LCV localization of their host target LULL1. (a) Representative confocal images show the localization of LULL1-HA (green) in HEK293T cells challenged with specified *Legionella* strains (red) for 1 hr. Scale Bar: 5 μm. (b) Quantitative analysis of LULL1-positive LCVs in cells infected with the indicated *Legionella* strains. Data were shown as means ± SEM of three independent experiments. At least 40 LCVs were counted for each condition.

**Figure 2.**
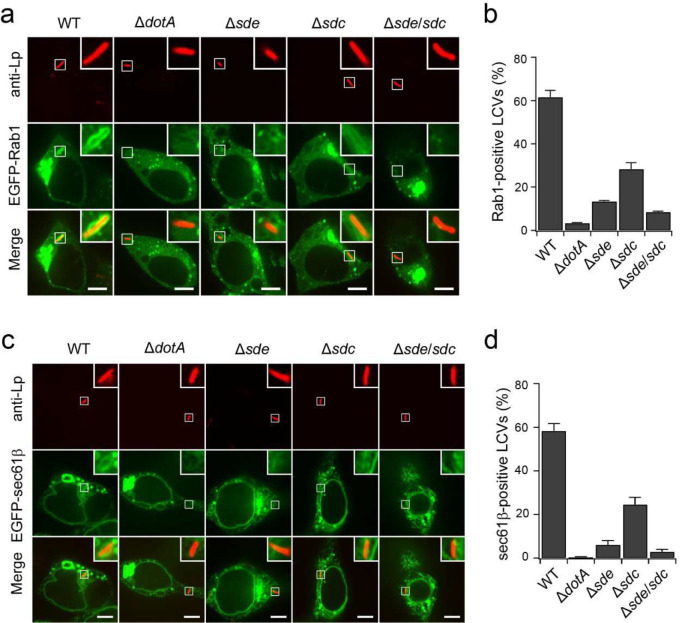
Sde and Sdc facilitate the recruitment of their host targets to the LCV. (a) Representative confocal images show the localization of EGFP-Rab1 (green) in HEK293T cells challenged with specified *Legionella* strains (red) for 1 hour. Scale Bar: 5 μm. (b) Quantitative analysis of Rab1-positive LCVs in cells infected with the indicated *Legionella* strains. Data were shown as means ± SEM of three independent experiments. At least 60 randomly selected LCVs were counted for each condition. (c) Representative confocal images show the recruitment of an ER-marker protein Sec61β (green) at the LCV (red). Scale Bar: 5 μm. (d) Quantitative analysis of Sec61β-positive LCVs. Data were shown as means ± SEM of three independent experiments. At least 60 randomly selected LCVs were counted for each condition.

Previous works indicated that Sdc and Sde were involved in recruiting ER membranes to the LCV ^28, 42, 44, 45^. We hypothesized that Ub-modifications of host targets by Sdc and Sde synergistically recruit their host targets and ER membranes, to the LCV. To test this hypothesis, we investigated the localization of host substrates and ER membrane markers in infected cells. HEK293T cells expressing FcγRII and EGFP-Rab1 were challenged with WT and mutant *Legionella* stains and the localization of Rab1 was analyzed by confocal microscopy at 1 hpi (Fig. 2a, b). In cells infected with the WT strain, ~61% of the LCVs were detected positive for Rab1, while Rab1-positive LCVs were largely reduced in cells infected with the Δ*sde* (~13%) or Δ*sdc* (~29%) strain. More strikingly, as low as 8% of LCVs were positive for Rab1 in cells infected with the Δ*sde*/*sdc* strain. Similar patterns were also observed for another confirmed Sdc and Sde target, LULL1 (extended data Fig. 3). These results suggest that the LCV-recruitment of host substrates depends on the activity of both Sdc and Sde families of effectors. We next analyzed the role of Sdc and Sde in the recruitment of ER membranes to the LCV using the ER membrane marker Sec61β (Fig. 2c, d). HEK293T cells expressing FcγRII and Sec61β were infected with *Legionella* strains and the recruitment of Sec61β to the LCV is examined by confocal microscopy at 2 hpi. In cells infected with WT *Legionella*, ~58% LCVs were positive for Sec61β, while in cells infected with the Δ*sde*, Δ*sdc*, or Δ*sde*/*sdc* strains, the percentage of Sec61β-positive LCVs dropped to 6%, 24%, and 3%, respectively. These observations collectively indicate that Sdc and Sde family proteins are both essential for the efficient recruitment of host target proteins and ER membranes to the LCV.

### Sdc and Sde build mixed Ub chains on host targets

**Figure 3.**
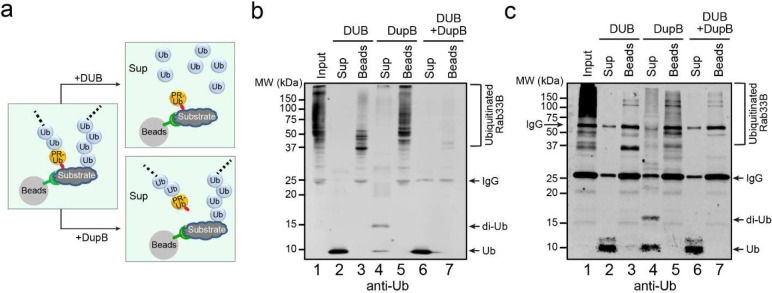
Sde and Sdc synthesize mixed ubiquitin chains on their host targets. (a) A schematic presentation of the mixed Ub chains on a substrate and the predicted outcomes after DUB or DupB cleavage. (b) Immunoblot image of in vitro ubiquitinated Rab33B after the cleavage by DUB and/or DupB. Purified recombinant 4xFlag-Rab33B was first ubiquitinated by SidC and SdeA enzymes in vitro and then immobilized on anti-Flag resins followed by the cleavage with DUB and/or DupB. The cleaved products released in the supernatant (Sup) and remained on the beads (Beads) were analyzed by SDS-PAGE followed by anti-Ub Western blot. (c) Immunoblot analysis of Rab33B prepared from infected cells after the cleavage by DUB and/or DupB. HEK293T cells expressing FCγRII and 4xFlag-Rab33B were infected with wild-type *Legionella* strain for 2 hours. 4xFlag-Rab33B was enriched by anti-Flag resins followed by the treatment with DUB and/or DupB. The cleaved products that were released in the supernatant (Sup) and that remained on the beads (Beads) were analyzed by SDS-PAGE followed by anti-Ub Western blot.

The finding that Sdc and Sde work together to modify the same host substrate triggered us to wonder about the nature of the poly-Ub chains conjugated to host targets by Sdc and Sde. Since the Sde effectors attach mono-PR-Ub to serine residues located at flexible loops of substrate proteins ^29–32^, and the Sdc effectors catalyze canonical ubiquitination on lysine residues of substrates or a pre-conjugated Ub molecule ^42^, we hypothesized that Sdc and Sde work together to synthesize mixed Ub chains on host proteins (Fig. 3a). In this model, the Sde effectors attach a PR-Ub moiety to serine residues of the substrate while the Sdc effectors conjugate poly-Ub chains either directly to substrate lysine residues or lysine residues of the pre-conjugated PR-Ub moiety. Based on this model, we predicted that complete cleavage of the ubiquitinated substrate by a canonical DUB will yield free mono-Ub molecules in the solution while PR-Ub will remain attached to the substrate. On the other hand, treatment with DupB will specifically detach PR-Ub from the substrate and thus will release poly-Ub chains with a variable length while canonical poly-Ub chains will remain conjugated to the substrate (Fig. 3a).

**Extended Data Figure 4.**
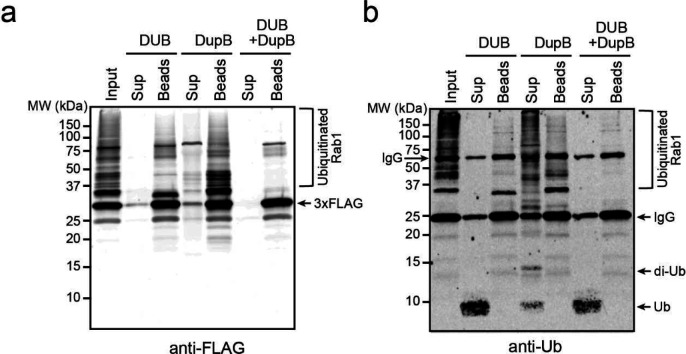
Immunoblot analysis of Rab1 prepared from infected cells after the cleavage by DUB and/or DupB. (a) HEK293T cells expressing FCγRII and 3xFlag-Rab1 were infected with wild-type *Legionella* strain for 2 hours. 3xFlag-Rab1 was enriched by anti-Flag resins followed by the treatment with DUB and/or DupB. The cleaved products that were released in the supernatant (Sup) and that remained on the beads (Beads) were analyzed by SDS-PAGE followed by anti-Flag, and (b) anti-Ub Western blot.

To test this hypothesis, we performed the DUB/DupB treatment experiments on ubiquitinated samples prepared both in vitro and in vivo. For the in vitro experiment, recombinant 4xFlag-Rab33B was ubiquitinated and PR-ubiquitinated by both the catalytic domain of SidC and SdeA. Rab33B was immobilized on anti-Flag resins and then treated with either the DUB or DupB. After the treatment, the supernatants were separated from the beads and both the supernatant and the bead fractions were analyzed by Western blot (Fig. 3b). As predicted, DUB treatment yielded predominantly mono-Ub in the supernatant while PR-Ub remained attached to the substrate (Fig 3b, lanes 2 and 3). On the other hand, after DupB treatment, Ub chains with various lengths were released in the supernatant while the substrate remained partially poly-ubiquitinated (Fig 3b, lanes 4 and 5). However, nearly all Ub moieties were released as mono-Ub from the substrate after the treatment with both the DUB and DupB (Fig. 3b. lanes 6 and 7). Similar results were obtained by the analysis of ubiquitinated Rab33B (Fig. 3c) and Rab1 (Extended data Fig. 4) samples prepared from cells challenged with WT *L. pneumophila*. Taken together, these results demonstrated that Sdc and Sde work together to build mixed Ub chains on host substrates. The Sdc effectors likely synthesize poly-Ub chains either directly to host substrates or to the PR-Ub moieties conjugated to the substrate by Sde.

### The poly-Ub chains synthesized by Sdc and Sde prevent the recognition by autophagy adaptor p62

**Extended Data Figure 5.**
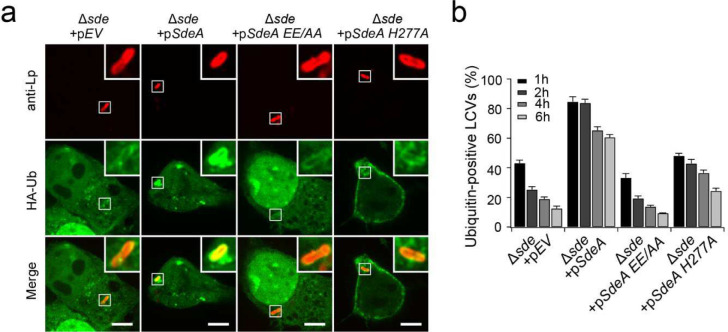
The enrichment of ubiquitinated species at the LCV in cells infected with the Δ*sde* strain supplemented with plasmids expressing WT and mutant SdeA. (a) Representative confocal images showing Ub signals (green) at the LCV in HEK293T cells challenged with indicated *Legionella* strains (red) at 1 hpi. Scale Bar: 5 μm. (b) Quantitative analysis of Ub-positive LCVs in cells infected with the indicated *Legionella* strains. Data were shown as means ± SEM of three independent experiments. At least 60 randomly selected LCVs were counted for each condition.

**Figure 4.**
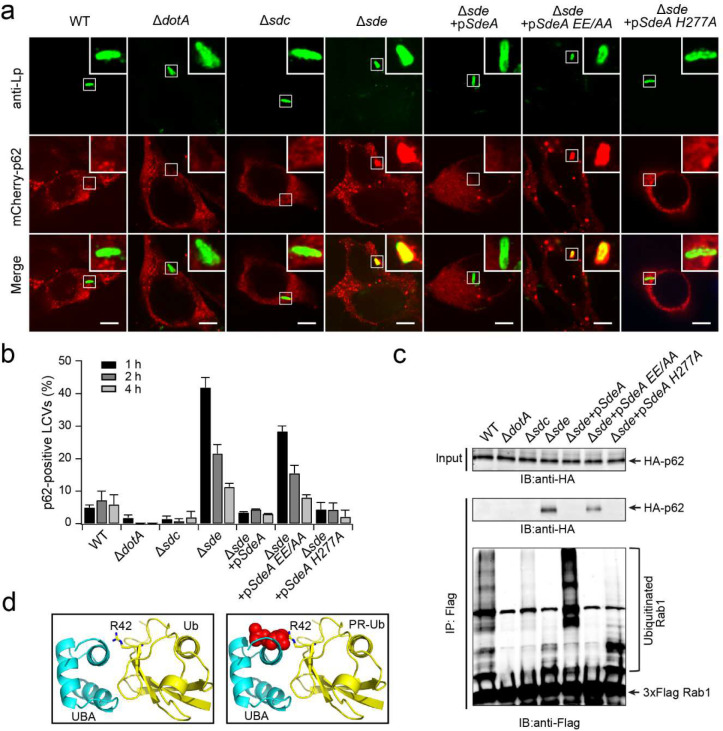
Sde suppresses the recruitment of p62 to the LCV by disrupting the physical interaction between p62 and Ub. (a) Representative confocal images show the localization of mCherry-p62 (red) in HEK293T cells challenged with specified *Legionella* strains (green) for 1 hour. Scale Bar: 5 μm. (b) Quantitative analysis of p62-positive LCVs in cells infected with the indicated *Legionella* strains. Data were shown as means ± SEM of three independent experiments. At least 40 randomly selected LCVs were counted for each condition. (c) Co-immunoprecipitation assay of the interaction between p62 and ubiquitinated Rab1. HEK293T cells expressing FcγRII, 3xFlag-Rab1, and HA-p62 were infected with the indicated *Legionella* strains for 1 hour. 3xFlag-Rab1 was enriched by anti-Flag immunoprecipitation. Immunoprecipitated materials were analyzed by anti-Flag (Rab1) and anti-HA (p62) Western blot. (d) Ribbon diagrams of the UBA-Ub complex (PDB ID: 2g3q) and the modeled UBA-PR-Ub complex. The UBA domain is colored in blue and the Ub is in yellow. The PR group attached to Ub R42 is shown in red spheres. Note the steric collision between the UBA domain and the PR group. This figure was generated using PyMol.

The LCV was enriched with ubiquitinated species at both early and late time points of the infection cycle, however, previous studies showed that Ub-binding autophagy adaptors were excluded from the LCV, and the exclusion depended on the Sde family members ^36^. To investigate the mechanism of the avoidance of autophagy adaptor recognition by the Ub-positive LCV, we examined p62 recruitment at the LCV in *Legionella*-challenged cells and the interaction between p62 and host proteins ubiquitinated by Sdc and Sde. HEK293T cells expressing FcγRII and mCherry-p62 were infected with *L. pneumophila* strains. Although most LCVs were positive for Ub signals at 1 hpi (Fig. 1a, b), a very low percentage (~4 %) of LCVs were detected positive for p62 (Fig. 4a, b). Almost no LCVs were positive for p62 in cells infected with the Δ*dotA* or Δ*sdc* strain. However, in cells challenged with the Δ*sde* strain, up to 40% of the LCVs were positive for p62 at 1 hpi (Fig. 4a,b) although the percentage of LCVs positive for Ub was reduced by more than 2-fold compared to the infection with the WT strain (Fig 1a,b). The p62-positive vacuoles decreased at later infection times (Fig. 4b) correlating with decreased Ub signals at the LCV (Fig. 1b and extended data Fig. 5). The ability to suppress p62 recruitment to the LCV was fully restored when the Δ*sde* strain was supplemented with a plasmid expressing WT SdeA (Fig. 4a,b) regardless of the high percentage (>80%) of Ub-positive LCVs (extended data Fig. 5). Surprisingly, the ability to suppress p62 recruitment to the LCV was also fully restored when the Δ*sde* strain was introduced to express the SdeA_H277A_ mutant, which was able to ADP-ribosylate Ub but was defective in PR-Ub ligation, but not the SdeA_EE/AA_ mutant, which was defective in its mART activity (Fig. 4a, b). It is worth noting that cells infected with the Δ*sde+*p*SdeA*_*H277A*_ strain exhibited a higher percentage of Ub-positive LCVs (48%) than with the Δ*sde*+p*SdeA*_*EE/AA*_ strain (33%) after 1 hour of infection (extended data Fig. 5) indicating that p62 recruitment does not correlate with the amount of Ub species at the LCV. Together, these results let us hypothesize that the observed suppression of p62 recruitment to the LCV is likely attributed to the failure of the direct binding of p62 with the ubiquitinated species generated by Sdc and Sde.

**Extended Data Figure 6.**
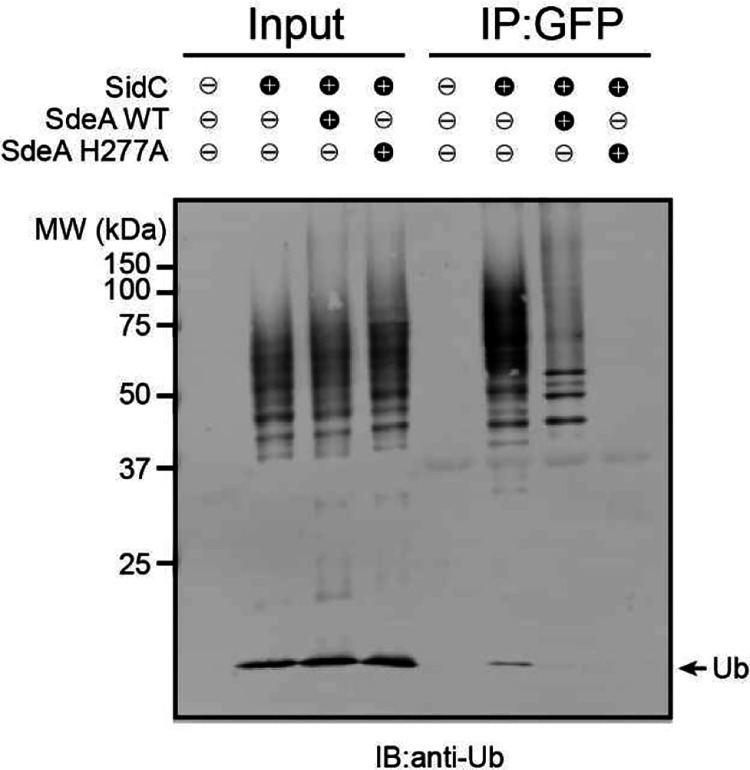
Pull-down assay of the interaction between the UBA_p62_ domain and in vitro ubiquitinated Rab33B. Recombinant 4x-Flag Rab33B was ubiquitinated by the catalytic domains of SidC and/or SdeA. The products were incubated with GFP-UBA_p62_ and precipitated by anti-GFP nanobody conjugated resins. The input and pull-down samples were analyzed by SDS-PAGE followed by anti-Ub Western blot.

To test this hypothesis, we examined the interaction between p62 and ubiquitinated substrates that were enriched from cells infected with *L. pneumophila*. HEK293T cells expressing FcγRII, 3xFlag-Rab1, and HA-p62 were challenged with *Legionella* strains for 1 hour. 3xFlag-Rab1 was immunoprecipitated with anti-Flag resins and the immunoprecipitated materials were analyzed by Western blot. In agreement with the p62 recruitment assay (Fig. 4a), HA-p62 was co-immunoprecipitated with the Rab1 proteins from cells infected with the Δ*sde* strain or the Δ*sde* strain supplemented with a plasmid expressing the SdeA_EE/AA_ mutant, but not the WT, Δ*dotA,* or the Δ*sdc* strain introduced to express either WT SdeA or the SdeA_H277A_ mutant (Fig. 4c). Similar results were obtained by an in vitro pull-down assay (Extended data Fig. 6). GFP-fused recombinant proteins of the Ub-associated (UBA) domain of p62 were purified and immobilized to GFP-nanobody resins to pull down ubiquitinated Rab33B. Rab33B proteins ubiquitinated by SidC alone were effectively pulled down by GFP-UBA_p62_, however, the interaction between GFP-UBA_p62_ and Rab33B ubiquitinated by both SidC and SdeA was markedly reduced. More strikingly, the interaction was nearly completely abolished when the Rab33B proteins were ubiquitinated by both SidC and the SdeA_H277A_ mutant (Extended data Fig. 6). These data confirmed that the poly-Ub chains synthesized by Sdc and Sde are insusceptible to the recognition by autophagy receptor p62. This observation allowed us to postulate that the disruption of the interaction between the UBA_p62_ domain with poly-Ub chains may be caused by the modification at Ub R42 with a phosphoribose (PR) (by WT SdeA) or an ADP-ribose (ADPR) group (by SdeA_H277A_ mutant). In fact, it has been shown that the UBA domain binds Ub at the I44 hydrophobic patch, with R42 in its proximity ^46^. The presence of PR or ADPR modification on R42 will likely cause steric collision against the binding with the UBA_p62_ domain (Fig. 4d).

### The poly-Ub chains synthesized by Sdc and Sde are phosphoribosylated by Sde

**Extended Data Figure 7.**
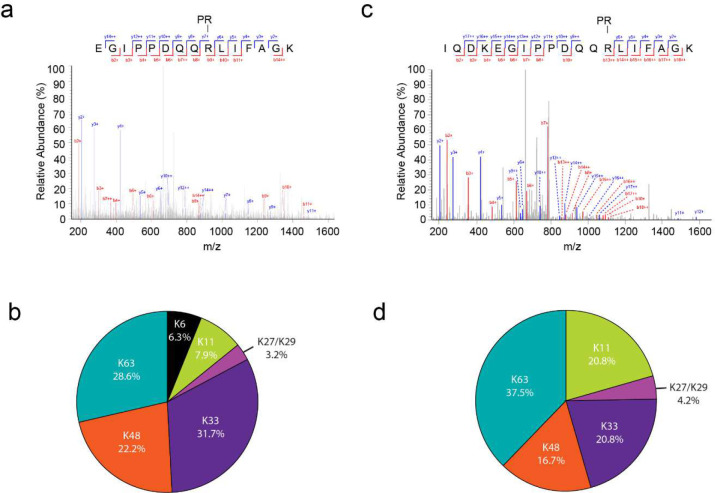
Ubiquitin modification and linkage analysis of Sde and Sdc modified substrates. (a) LC-MS/MS spectrum of a Ub peptide from an in vitro ubiquitinated sample demonstrating the phosphoribosyl modification of R42. Recombinant 4x-Flag Rab33B was ubiquitinated in vitro by SidC and SdeA and subsequently enriched by anti-Flag beads. The resulting bound proteins were eluted and analyzed by LC-MS/MS. (b) Pie chart corresponding to in vitro ubiquitin linkage analysis. Samples prepared in (a) were further analyzed to detect diglycine-modified peptides (K-GG) generated following trypsin digestion. The percentages in the pie chart correspond to the occurrence of each ubiquitin linkage identified during the analysis. (c) LC-MS/MS spectrum of a Ub peptide from a sample prepared from *Legionella* infected cells. HEK293T cells expressing FCγRII and EGFP-3x-Flag Rab1 were infected with WT strain for 2 hours. EGFP-3xFlag-Rab1 was immunoprecipitated and samples were prepared similarity as in (a). (d) Pie chart corresponding to in vivo ubiquitin linkage analysis.

**Figure 5.**
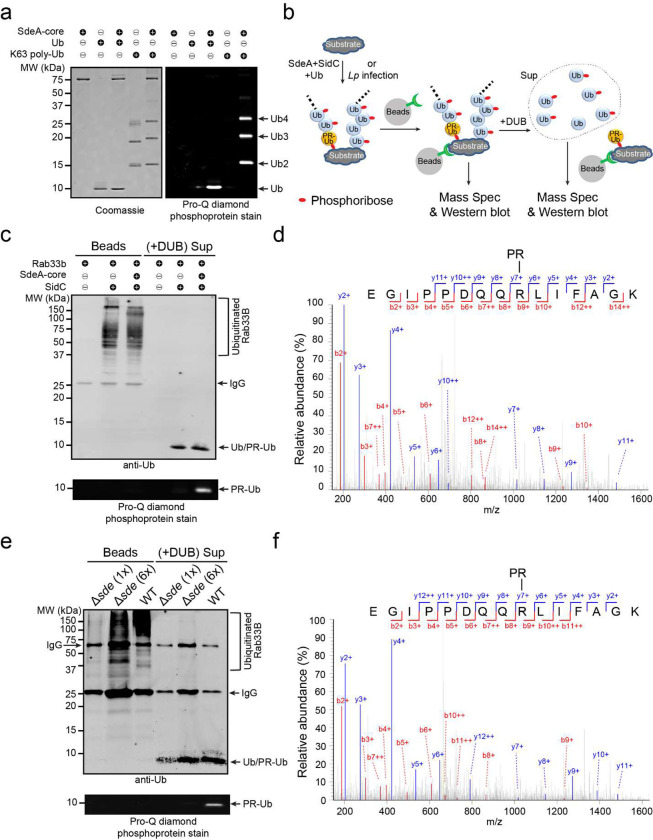
Sde modifies poly-Ub chains by phosphoribosylation. (a) In vitro modification of K63-linked poly-Ub chains by SdeA. Mono-Ub or K63-linked poly-Ub chains were incubated with the catalytic core of SdeA and NAD^+^. The products were analyzed by SDS-PAGE followed by staining with Coomassie blue (Left panel) or Pro-Q diamond (Right panel). (b) Schematic diagram of the poly-Ub chains attached to the substrate and experimental flow of biochemical and Mass spectrometry analysis of the poly-Ub chains. (c) Biochemical analysis of Ub modification by Sde in vitro. Recombinant 4x-Flag-Rab33B was ubiquitinated in vitro by the catalytic domain of SidC or by both SidC and SdeA. Ubiquitinated Rab33B was immobilized on anti-Flag beads and treated with a canonical DUB. The samples before and after the DUB treatment were analyzed by SDS-PAGE followed by anti-Ub Western blot (top panel) or by Pro-Q diamond stain (bottom panel). (d) LC-MS/MS spectrum of a Ub peptide from an in vitro ubiquitinated sample showing the phosphoribosyl modification at Ub R42. Recombinant 4x-Flag Rab33B was ubiquitinated in vitro by SidC and SdeA and then enriched by anti-Flag beads. The resulting bound proteins were treated with a purified DUB, and the Ub molecules released from the cleavage were analyzed by LC-MS/MS. (e) Biochemical analysis of Ub modification by Sde in vivo. HEK293T cells expressing FCγRII and 4xFlag-Rab33B were infected with WT or Δ*sde* strain for 2 hours. 4xFlag-Rab33B was immunoprecipitated by anti-Flag resins and then treated with a canonical DUB. The samples before and after the DUB treatment were analyzed by SDS-PAGE followed by anti-Ub Western blot (top panel) or by Pro-Q diamond stain (bottom panel). Samples prepared from cells infected with the Δ*sde* strain were loaded with 1 fold (1x) or 6 folds (6x) of the amount of the sample from the WT infection. (f) LC-MS/MS spectrum of a Ub peptide from a sample prepared from *Legionella* infected cells showing the phosphoribosyl modification at Ub R42. Samples were prepared similarly as in (e).

To examine whether poly-Ub chains can be modified by Sde, we analyzed the modification on Ub using both biochemical and mass spectrometry approaches. First, K63-linked poly-Ub chains were treated with SdeA Core-WT, which contains functional mART and PDE domains. The phosphoribosyl modification on Ub moieties can be detected by SDS-PAGE followed by Pro-Q diamond phosphoprotein stain (Fig. 5a), which specifically stains exposed phosphoryl groups ^47^. This result demonstrated that free poly-Ub chains can be phosphoribosylated by Sde. Next, we asked whether poly-Ub chains attached to a substrate by Sdc and Sde are also modified with phosphoribose. To answer this question, we prepared ubiquitinated substrates both in vitro using recombinant SidC and SdeA enzymes and in vivo from *Legionella*-infected cells. The ubiquitinated substrates were either directly analyzed by MS/MS or digested with a canonical DUB (the N-terminal domain of SdeA) followed by MS/MS analysis of the released free Ub (Fig. 5b). For the in vitro experiment, recombinant 4xFlag-Rab33B was ubiquitinated by SidC or by both SidC and SdeA. Ubiquitinated Rab33B was then treated with DUB and the supernatant samples were separated by SDS page and analyzed by anti-Ub Western blot and Pro-Q diamond phosphoprotein stain. PR-Ub was only detected in the supernatant from the sample ubiquitinated by both SidC and SdeA but not by SidC alone (Fig. 5c). Phosphoribosyl modification on Ub moieties within the Ub chains synthesized by SidC and SdeA in vitro was further verified by MS/MS analysis. An additional mass of 201.009 Da, corresponding to a phosphoribosyl group was detected at Ub R42 (Fig. 5d). We next examined the modification on Ub moieties from poly-ubiquitinated substrates under infection conditions (Fig. 5e). FcγRII and 4xFlag-Rab33B-expressing HEK293T cells were infected with WT or Δ*sde Legionella* strains for 2 hours. Ubiquitinated Rab33B was immunoprecipitated with anti-Flag resins. Following a similar procedure described above, PR-Ub molecules were only detected from samples originating from cells infected with the WT but not the Δ*sde* strain (Fig. 5e, f). Phosphoribosyl modification can also be detected in both in vitro and in vivo ubiquitinated samples by MS/MS without the DUB treatment (Extended data Fig. 7a and c). Interestingly, we also detected Ub chain linkages in these samples. In agreement with the previously reported results ^42^, we found that SidC prefers to catalyze K63, K48, and K33 Ub chains (Extended data Fig. 7b and d). Taken together, our results demonstrated that the Ub moieties within the poly-Ub chains synthesized by Sdc and Sde are phosphoribosylated by Sde.

### The PR-Ub-specific deubiquitinases, DupA and DupB can process ADPR-Ub to PR-Ub on internal Ub moieties of poly-Ub chains

**Figure 6.**
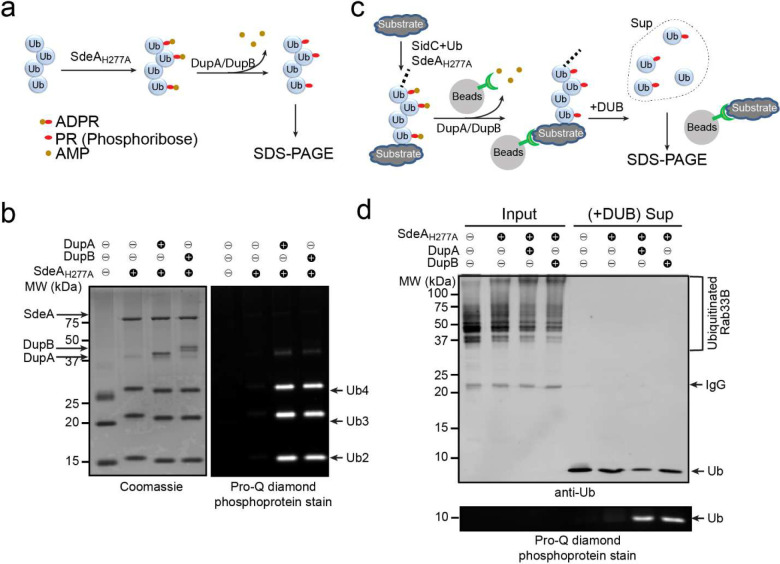
Both DupA and DupB play a role in the processing of ADPR-Ub to PR-Ub. (a) Schematic representation of DupA and DupB modification on free poly-Ub chains. (b) K63-linked poly-Ub chains were first modified by purified SdeA_H277A_ and subsequently treated with DupA or DupB. Conversion of ADPR-Ub to PR-Ub following DupA or DupB treatment was analyzed by SDS-PAGE followed by Coomassie blue (left panel) or Pro-Q diamond stain (right panel). (c) Schematic representation of DupA and DupB modification on a poly-ubiquitinated substrate. (d) 4x-Flag Rab33B was poly-ubiquitinated by recombinant SidC and modified by SdeA_H277A_. Ubiquitinated Rab33B was immobilized on anti-Flag beads and then treated with DupA or DupB. The samples were then treated with a canonical DUB to release mono-Ub. Samples before and after DUB cleavage were analyzed by SDA-PAGE followed by anti-Ub Western blot (top panel) or by Pro-Q diamond stain (bottom panel).

The phosphoribosyl modification of Ub is a two-step reaction, of which the first step involves ADP-ribosylation on Ub by the Sde mART activity and the second step involves the hydrolysis of ADPR to PR with the releasing of an AMP molecule. Our previous results showed that several PDE domain-containing effectors, including the PR-Ub specific deubiquitinases, DupA and DupB, were able to process mono-ADPR-Ub to PR-Ub ^45^. We thus asked whether DupA and DupB contribute to the cleavage of ADPR to PR on poly-Ub chains. To answer this question, free poly-Ub chains were first ADP-ribosylated by SdeA-Core H227A mutant and then treated with either DupA or DupB (Fig. 6a). In contrast to the untreated, samples treated with either DupA or DupB exhibited positive signals upon Pro-Q Diamond stain, indicating that both DupA and DupB can cleave ADPR to PR that is attached to Ub moieties within the poly-Ub chain (Fig. 6b).

Beyond free poly-Ub chains, DupA and DupB were also capable of processing ADPR-Ub to PR-Ub on poly-ubiquitinated substrates. Ubiquitinated Rab33B was generated in the presence of SidC and SdeA-Core H227A and the product was then treated with either DupA or DupB. The Ub moieties were then released from the substrate by treatment with DUB and analyzed SDS-PAGE followed by Pro-Q Diamond stain (Fig. 6c). PR-Ub was detected from DupA or DupB-treated samples but not from untreated ones (Fig. 6d). Collectively, these results indicate that the previously identified PR-Ub specific deubiquitinases, DupA and DupB, may also be involved in the phosphoribosyl modification of poly-Ub chains by processing ADPR to PR.

### Cross-linking of multiple Sdc and Sde targets by canonical and PR-ubiquitination

**Extended Data Figure 8.**
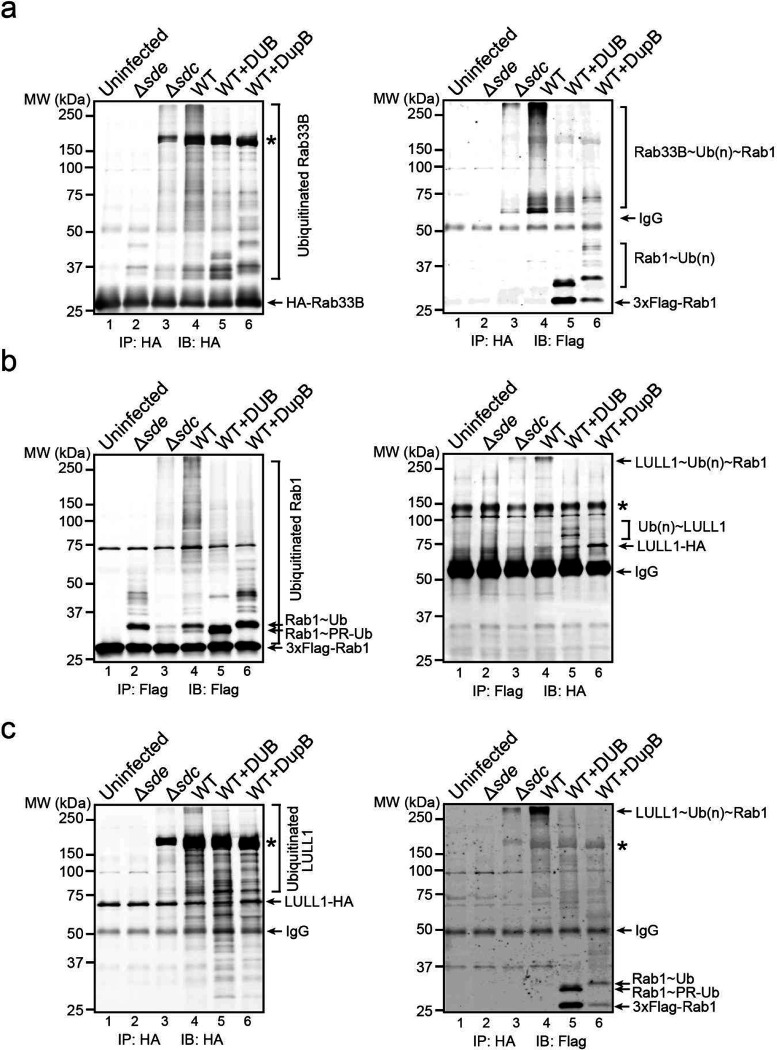
Cross-linking of multiple Sdc and Sde targets by canonical and PR-ubiquitination. (a) Immunoblot analysis of crossed-linked Rab1 and Rab33B in *Legionella* infection. HEK293T cells expressing FCγRII, 3xFlag-Rab1, and HA-Rab33B were infected with indicated *Legionella* strains for 2 hours. HA-Rab33B was immunoprecipitated by anti-HA resins and then treated with a canonical DUB or DupB. The samples before and after the treatment were analyzed by SDS-PAGE followed by anti-HA Western blot (left panel) or by anti-Flag Western blot (right panel). (b) Immunoblot analysis of crossed-linked Rab1 and LULL1 in *Legionella* infection. HEK293T cells expressing FCγRII, 3xFlag-Rab1, and LULL1-HA were infected with indicated *Legionella* strains for 2 hours. 3xFlag-Rab1was immunoprecipitated by anti-Flag resins and then treated with a canonical DUB or DupB. The samples before and after the treatment were analyzed by SDS-PAGE followed by anti-Flag Western blot (left panel) or by anti-HA Western blot (right panel). (c) Immunoblot analysis of anti-HA immunoprecipitated samples from the same cells lysates prepared as in (b). * non-specific signals.

**Figure 7.**
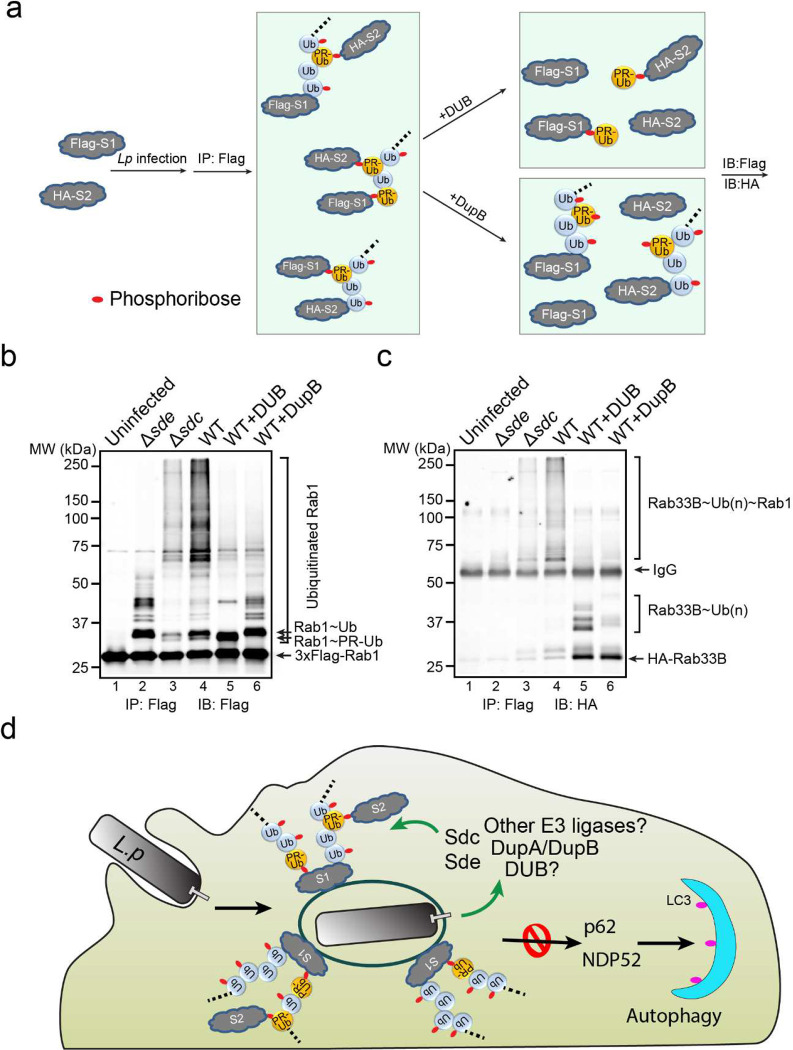
Cross-linking of multiple Sdc and Sde targets by canonical and PR-ubiquitination. (a) Schematic model of two host substrates that are cross-linked by canonical and PR-ubiquitination and the experimental flow of verification. In this model, a poly-Ub chain attached to one substrate (either through canonical or PR-ubiquitination) and the Ub moieties within the Ub chain can be used to modify a second substrate via PR-ubiquitination. The cleavage by a canonical DUB will result in unmodified and mono- or multi-mono PR-ubiquitinated products, while the cleavage by DupB will yield unmodified and ubiquitinated (with a variable length) products. (b) and (c) Biochemical analysis of substrate crossing-linking in *Legionella* infection. HEK293T cells expressing FCγRII, 3xFlag-Rab1, and HA-Rab33B were infected with indicated *Legionella* strains for 2 hours. 3xFlag-Rab1 was immunoprecipitated by anti-Flag resins and then treated with a canonical DUB or DupB. The samples before and after the treatment were analyzed by SDS-PAGE followed by anti-Flag (b) or anti-HA Western blot (c). (d) A schematic model of unconventional poly-Ub chains at the LCV serving as a scaffold for cross-linking multiple host targets and preventing host autophagy detection.

The Sde family effectors conjugate mono-Ub to a substrate through a two-step reaction, namely PR-ubiquitination. Now we showed that Sde effectors also ADP-ribosylate internal Ubs within the poly-Ub chains. Although some of the ADPR groups can be further processed into PR by Sde or DupA/DupB, it is legitimate that some of the internal Ubs of the poly-Ub chain attached to one substrate can also be conjugated to a second substrate through PR-ubiquitination by Sde. In this way, multiple host targets can be covalently cross-linked to one poly-Ub chain created by Sdc and Sde (Fig. 7a). To test this hypothesis, we infected cells expressing two known Sdc and Sde substrates, 3xFlag-Rab1 and HA-Rab33B, with WT or mutant *Legionella* strains. Cell lysates were prepared after 2 hpi followed by immunoprecipitation with anti-Flag resins and analyzed by both anti-Flag (Fig. 7b) and anti-HA (Fig. 7c) Western blot. High molecular weight bands positive for both Rab1 (Fig. 7b, lane 4) and Rab33B (Fig. 7c, lane 4) were detected in samples from cells infected with the WT strain, but not from uninfected cells or cells infected with Δ*sde* (Fig. 7b, c, lanes 1 and 2). These high molecular weight bands were markedly reduced in samples from cells infected with the Δ*sdc* strain (Fig. 7b, c, lane 3). These observations suggest that Rab33B and Rab1 were cross-linked to form the high molecular weight species allowing the co-immunoprecipitation of Rab33B with Rab1. To elucidate the nature of the cross-linking, the immunoprecipitated samples from cells infected with the WT strain were treated with DUB or DupB and analyzed by Western blot. As expected (Fig. 7a), after DUB treatment, the high molecular weight bands corresponding to cross-linked species were eliminated and resulted in unmodified and PR-ubiquitinated (mono- or multiple mono-) Rab1 (Fig. 7b, lane 5) and Rab33B (Fig. 7c, lane 5). Accordingly, DupB treatment resulted in unmodified and poly-ubiquitinated (with variable lengths) Rab1 (Fig. 7b, lane 6) and Rab33B (Fig. 7c, lane 6). Cross-linking between Rab1 and Rab33B was further verified using samples prepared by revered immunoprecipitation with anti-HA resins (Extended data Fig. 8a). Furthermore, cross-linking of Sdc and Sde substrates in *Legionella* infection was also observed between other host targets (for example, between Rab1 and LULL1, Extended data Fig. 8b and c). Together, these results demonstrated that multiple host targets were cross-linked through unconventional Ub chains that were generated by Sde and Sdc at the LCV.

## Discussion

In this study, we reported a coordinated interplay between two families of Ub-hijacking effectors, the Sdc family effectors, which mimic host E3 ligases and catalyze canonical poly-ubiquitination, and the Sde effectors, which catalyze unconventional phosphoribosyl-linked serine ubiquitination (PR-ubiquitination) on host targets. Our data support a model of poly-Ub chains built by these two families of effectors around the LCV (Fig. 7d). In this model, the translocated Sdc ligases catalyze canonical poly-ubiquitination either directly to host proteins or to a PR-Ub molecule that is attached to the same host protein by Sde. Furthermore, the Sde effectors modify the Ub moieties within the poly-Ub chain via ADP-ribosylation and the attached APD-ribose group can be further processed into phosphoribose (PR) by the PDE domain of Sde or DupA/DupB, two previously identified PR-Ub specific deubiquitinases. Interestingly, instead of being hydrolyzed to PR, the internal ADP-ribosylated Ub moieties can also be conjugated to other host substrates through PR-ubiquitination by the PDE domain of Sde. In this way, two or more Sdc and Sde substrates can be conjugated to the same poly-Ub chain (Fig. 7d). Thus a “Ub-coat” containing unconventionally modified Ub chains was built around the LCV by the *Legionella* bacterium.

An apparent paradox associated with the “Ub-coat” at the LCV is that the Ub signals do not trigger autophagy clearance of the bacterium. It has been postulated that potential *cis*-acting effectors may modify the “Ub-coat” and hence disrupt the recruitment of autophagy adaptors to the LCV ^36^. Surprisingly, a recent publication reported that the exclusion of p62 from the LCV was due to Sde-mediated PR-ubiquitination of host Ubiquitin-Specific Protease 14 (USP14), which disrupted the direct interaction between USP14 and p62 ^48^. However, this report ignored the fact that the LCV surface is enriched with ubiquitin species ^21, 22^ and the fact that ubiquitin chains are the major driving force for the recruitment of autophagy adaptors, such as p62 ^8, 49^. USP14 knockdown could not explain the exclusion of p62 from the Ub-enriched LCV. More importantly, our data showed that p62 was excluded from the LCV when host cells were infected with the Δ*sde+pSdeA*_H277A_ strain, which is unable to PR-ubiquitinate any host targets but only modifies Ub by ADP-ribosylation (Fig. 4a–c). Thus, PR-ubiquitination of host substrates per se does not account for the exclusion of p62, and the true mechanism underlying the avoidance of autophagy adaptors by the LCV remains to be elucidated. Here we provided direct evidence to support the “Ub-coat” modification model. We showed that the Sde family effectors are the *cis* factors to modify the Ub moieties within the Ub chains and Sde modification results in the disruption of the Ub adaptor-interacting surface on Ub molecules and hence prevents the recruitment of host autophagy adaptors to the LCV. This model is further supported by the results reported in the concomitantly submitted manuscript by Kotewicz et al. ^50^

Our studies revealed the structural nature of the unconventional poly-Ub chains created at the LCV. However, it is intriguing to contemplate how the “Ub-coat” around the LCV benefits *Legionella* infection. It has been reported that as soon as 15 min after bacterial uptake, the LCV is covered by ER-derived vesicle and enriched with ER residential proteins ^51, 52^. The recruitment of ER vesicles has been attributed to the manipulation of host small-GTPases, such as Rab1 ^53^, Rab33B, and Rab6A ^54^, by *Legionella* effectors. Here we showed that both Sdc and Sde effectors are required for the recruitment of their host targets and ER markers Sec61β (Fig. 2) and HDEL ^42^ to the LCV, suggesting canonical ubiquitination and PR-ubiquitination are critical in the accumulation of ER materials at the LCV. One explanation for ER vesicle recruitment to the LCV is that Sdc and Sde modify key host factors that control Golgi-to-ER trafficking, which causes altered functions of these host factors and results in the association of the LCV with the ER. However, given the fact that many ER proteins were found to be PR-ubiquitinated after *Legionella* infection ^44, 45^ and our new data showing the cross-linking of multiple host substrates to the poly-Ub chains generated by Sdc and Sde, the association of ER-derived vesicles with the LCV can be explained by an alternative mechanism. It is possible that the poly-Ub chains synthesized by Sdc and Sde provide multiple anchoring points to covalently crosslink proteins associated with either the LCV or ER vesicles and thus to tether together these two types of membrane bound vesicles. To further unravel the physiological role of the Ub-coat at the LCV is certainly an exciting future direction.

In this study, we reported that the Sdc and Sde families of effectors contribute to the creation of poly-Ub chains at the LCV. Furthermore, we also provided evidence to support that the PR-Ub-specific deubiquitinases, DupA and DupB, may also be involved in the poly-Ub chain assembly by processing ADPR to PR. However, *Legionella pneumophila* encodes a large number of effectors that hijack the host Ub system ^55–59^ and it is intriguing to ask whether other *Legionella* Ub-hijacking effectors are involved in the building of the “Ub-coat”. It is notable that when cells were infected with the Δ*sdc* strain, ubiquitinated Rab1 was detected even after the treatment with DupB (Fig. 1e), indicating Rab1 can also be ubiquitinated by other E3 ligases. However, the ubiquitin signals at the LCV were significantly reduced in cells infected with the Δ*sdc* strain (Fig. 1a and b), suggesting Sdc effectors are the major E3 ligases for the building of the “Ub-coat” at the LCV. Besides E3 ligases, *Legionella pneumophila* also encodes several effectors that function as a canonical DUB ^25, 60–65^. At least some of these DUBs have been shown to regulate overall ubiquitination at the LCV ^25, 60, 62, 63^. Interestingly, the N-terminal DUB domain of Sde family of effectors also contribute to the Ub species at the LCV ^50^. In this study, although we showed that the Sdc and Sde families of Ub ligases are the major players, other E3 ligases and DUBs are also involved in either the establishment or the dynamics of the “Ub-coat” at the LCV. Nevertheless, elucidating the physiological roles of each player in the “Ub-coat” requires more delicate experiments in the future.

In summary, we presented the results to show two distinct family Ub E3 ligases, Sdc and Sde work together to assemble a “Ub-coat” at the LCV during Legionella infection. This “Ub-coat” contains unconventional mixed Ub chains with internal Ub moieties being modified with a phosphoribosyl group. This modification is important for preventing the recognition by p62. Our results elucidate the nature of the “Ub-coat” and provide a molecular mechanism for the avoidance of autophagy adaptors and therefore the protection of the LCV from xenophagy clearance.

## Materials and Methods

### Cloning and Mutagenesis.

For expression in mammalian cells, EGFP-Rab1, 4xFlag-Rab33B, LULL1-HA and HA-Ub were generated as previously described ^24, 45^. To generate 3xFlag-Rab1, Rab1 was amplified from EGFP-Rab1, digested with EcoRI and BamHI and inserted into pCMV 3xFlag 7.1 vector digested with the same restriction enzymes. To generate EGFP-3xFlag-Rab1, 3xFlag-Rab1 fragment was amplified, digested with BamHI and XhoI, then inserted into pEGFP-C1 vector digested with BglII and SalI. To generate EGFP-sec61β, a fragment of EGFP was PCR amplified, digested with NheI and BspEI, and then inserted into pTagBFP-sec61β obtained from Dr. Fenghua Hu (Cornell University) and digested with the same restriction enzymes. HA-p62 and mCherry-p62 were also obtained from Dr. Fenghua Hu (Cornell University). Flag-Syntaxin 17 was purchased from Addgene (Addgene #19506).

For *Legionella* expression, DNA fragments of wild-type and mutants of SidC and SdeA were amplified from plasmids for mammalian expression ^24, 45^, and digested with BamHI and XhoI. The digested DNA fragment was inserted into pZL507 plasmid obtained from Dr. Zhao-Qing Luo (Purdue University) and digested with BamHI and SalI. To delete sdcA-sidC genes in Δ*sde* strain, DNA fragments of 1.2 Kb upstream and 1.2 Kb downstream of the *sdc* (*sdcA-sidC*) gene loci were PCR amplified and cloned into the pSR47s vector obtained from Dr. Zhao-Qing Luo (Purdue University), to generate pSR47s-Δ*sdc* plasmid.

For protein expression in *E.coli*, SidC (aa.1–542), UbcH7, Ub WT and mutants, 4xFlag-Rab33B, DupA, DupB, human E1, His-TEV-Ub K6, Ubc13, and Mms2 were generated as previously described ^24, 45, 66, 67^. DNA fragments corresponding to the DUB domain of SdeA (aa.1–213) and SdeA-Core (aa.231–925) were PCR amplified from genomic DNA of *L. pneumophila* Philadelphia strain and digested with BamHI and XhoI. The corresponding DNA fragments were ligated into pET28a 6xHis-Sumo digested vector with the same restriction enzymes. Site-directed mutagenesis was performed with overlapping primers to generate the SdeA-Core H277A mutant. Constructs for 6xHis-Mms2, GST-Ubc13, Ub_K63R_, and Ub_D77_ were a gift from Dr. Scott Emr (Cornell University). To generate GFP-UBA_p62_, the UBA domain of p62 (aa. 382–436) was PCR amplified from mCherry-p62 and digested with BamHI and XhoI. The digested DNA fragment was inserted into pET28a 6xHis-GFP vector digested with the same restriction enzymes.

### Protein expression and purification.

All Bacterial expression constructs were transformed into *Escherichia coli* Rosetta (DE3) cells. Cultures from single colonies were grown in Luria-Bertani (LB) medium containing 50 μg/ml of kanamycin or 100 μg/ml of ampicillin to a density between 0.6 and 0.8 OD_600_. Protein expression was induced with 0.2 mM isopropyl-B-D-thiogalactopyranoside (IPTG) at 18°C overnight. Harvested cells were resuspended in lysis buffer (20 mM Tris-HCl pH 7.5, and 150 mM NaCl) and lysed by sonication. The lysates were clarified by centrifugation at 16,000 rpm for 45 minutes at 4°C, and the supernatant was incubated with cobalt resin (Gold-Bio; for His-tagged proteins) or glutathione Sepharose 4 Fast Flow resin (GE; for GST-tagged proteins) for 1.5 hours at 4°C. Bound proteins were washed extensively with lysis buffer. The SUMO-specific protease Ulp1 was added to the resin slurry to release the protein from the His-SUMO tag and resin. Eluted proteins were further purified by fast protein liquid chromatography (FPLC) size exclusion chromatography using either a Superdex S75 or S200 columns (GE Life Sciences). Peak fractions were then collected and concentrated. 6xHis-tagged GFP-UBA_p62_, cells were sonicated in lysis buffer (20 mM Tris-HCl pH 7.5 and 150 mM NaCl), cleared by centrifugation at 16,000 rpm for 45 minutes at 4°C, and incubated with cobalt resin for 1.5 hours at 4°C. Bound proteins were washed extensively and eluted with 250 mM imidazole in 20 mM Tris-HCl pH 7.5 and 150 mM NaCl. Proteins were further purified by size exclusion chromatography.

For Ub expression, purification protocols were adapted as previously described ^68^. Harvested cells were resuspended in lysis buffer (20 mM ammonium acetate pH 5.1 and 0.1 mM PMSF), lysed by sonication, and clarified by centrifugation at 16,000 rpm for 30 minutes at 4°C. The clarified lysate pH was lowered to 4.8 using glacial acetic acid and the solution was again centrifuged at 16,000 rpm for 30 minutes at 4°C. The pH of the remaining soluble fraction was adjusted to 5.1 with the addition of NaOH. The supernatant was loaded onto a HiTrap SP cation exchange column equilibrated in 20 mM ammonium acetate pH 5.1. Ubiquitin was eluted with a buffer gradient to 0.5 M ammonium acetate pH 5.1. Fractions corresponding to Ub were pooled and further purified by size exclusion chromatography in 20 mM Tris pH 7.5, and 150 mM NaCl. Final fractions containing ubiquitin were collected and concentrated.

Human E1 was purified as described previously ^66^. Briefly for 6xHis-Uba1, cells were harvested in lysis buffer (20 mM Tris pH 7.5, 150 mM NaCl, 1 mM PMSF, and cOmplete protease inhibitor) and lysed by sonication. After clarification, the supernatant was bound to cobalt resin, washed extensively, and eluted with 250 mM imidazole, 20 mM Tris pH 7.5, and 150 mM NaCl. Human E1 was further purified by reacting with ubiquitin conjugated to Affi-Gel 10. After incubation, the column was washed with 50 mM Tris-HCl pH 8.0 and 0.5 M KCl. Uba1 was eluted in 50 mM Tris-HCl pH 8.0 and 10 mM DTT, buffer exchanged to 50 mM Tris-HCl pH 8.0, 150 mM NaCl and further purified by size exclusion.

### Synthesis of K63 poly-Ub chains.

Prior to synthesis, 5x PBDM buffer was prepared with the following components: 250 mM Tris-HCl pH 8.0, 25 mM MgCl_2_, 50 mM creatine phosphate, 3 U/mL inorganic pyrophosphatase and 3 U/mL creatine phosphokinase. K63 linked poly-Ub was synthesized by incubating 0.1 μM human E1, 8 μM of Ubc13 and Mms2, 10 mg/mL WT-Ub, 5.6 mM ATP, 1x PDBM buffer, and 0.6 mM DTT. The reaction was incubated at 37 °C for 3 hours and then the reaction was quenched by 20-fold dilution into 50 mM ammonium acetate, pH 4.5. The sample was loaded onto a HiTrap SP cation exchange, and chains of defined lengths were separated using a linear gradient of 0–0.6 M NaCl. Peaks corresponding to di-Ub, tri-Ub, and tetra-Ub were pooled and the buffer was exchanged into 20 mM Tris-HCl pH 7.5, 150 mM NaCl and concentrated.

### *In vitro* synthesis of ubiquitinated Rab33B.

To synthesize canonically ubiquitinated Rab33B, 4 μM of purified 4x-Flag-Rab33B was mixed with 50 mM Tris-HCl pH 8.0, 1x PDBM, 2.5 mM ATP, 0.15 μM human E1, 0.2 μM UbcH7, 0.5 μM SidC (aa. 1–542), and 100 μM ubiquitin. The reaction was incubated at 37 °C for 1.5 hours. To synthesize mixed Ub chain-modified Rab33B, 4 μM 4x-Flag-Rab33B was incubated with 50 mM Tris-HCl pH 8.0, 1x PDBM, 2.5 mM ATP, 0.15 μM human E1, 0.2 μM UbcH7, 0.5 μM SidC (aa. 1–542), 1 μM SdeA-Core WT or SdeA-Core H227A mutant, 1 mM NAD^+^, and 100 μM ubiquitin at 37 °C for 2 hours. Ubiquitinated Rab33B was then purified with anti-Flag beads for DUB cleavage or other assays.

### In vitro UBA_p62_ binding assay.

Purified GFP-UBA_p62_ was first immobilized to GFP-nanobody conjugated resins and incubated with in vitro ubiquitinated Rab33B at 4°C for 2 hours. The resin was washed with a buffer containing 50 mM Tris, pH 8.0 for 3 times and materials bound to the resin were analyzed by SDS-PAGE followed by Western blot using an anti-Ub antibody.

### Poly-Ub chain modification assays.

Ubiquitination reactions were performed by mixing 1 μM WT SdeA-Core or Sde-Core H277A mutant with 12.5 μM K63 linked poly-Ub in a reaction buffer containing 50 mM Tris-HCl pH 7.5 and 50 mM NaCl, in the presence or absence of 1 mM NAD^+^. The reactions were incubated for 2 hours at 37 °C and reaction products were assessed by 15% SDS-PAGE stained with either Coomassie or Pro-Q Diamond phosphoprotein stain (Invitrogen). Only modified PR-Ub chains are visible by Pro-Q phosphoprotein stain due to its free phosphoryl group ^69^.

### DUB, DupA, and DupB cleavage assays.

The cleavage reactions were performed by adding 1 μM of DUB, DupA, or DupB to in vitro synthesized poly-Ub chains or ubiquitinated substrates at 37°C for 2 hours. For poly-Ub chain cleavage, the reaction products were assessed by 15% SDS-PAGE stained with either Coomassie or Pro-Q Diamond phosphoprotein stain (Invitrogen). For the cleavage of ubiquitinated substrates, the supernatants of the cleavage reactions were first collected by centrifugation at 6,000 rpm. The supernatant was concentrated by precipitation with the addition of PPT (0.1% glacial acetic acid, 49.9% ethanol, and 50% acetone) for 1 hour on ice. The protein pellets were resuspended in SDS loading buffer. The resins were washed 3 times with 50 mM Tris pH 8.0 and the remaining proteins attached to the resin were eluted with 1% SDS and 100 mM Tris pH 8.0. Samples were separated by 15% SDS-PAGE followed by Western blot using an anti-Ub antibody or stained with Pro-Q Diamond phosphoprotein stain.

### Cell culture and transfection.

HEK293T cells were cultured in Dulbecco’s modified Eagle’s medium (CellGro) supplemented with 10% fetal bovine serum (Gibco) and 1% penicillin-streptomycin (Invitrogen) at 37 °C with 5% CO_2_. Transfection was performed using a polyethyleneimine (PEI) reagent.

### *Legionella* strains and infection.

*L. pneumophila* strains used include the wild type Lp02 ^70^, the Dot/Icm deficient Lp03 ^70^, the Δ*sde* strain ^71^, the Δ*sdc* strain ^24^, and the Δ*sde/sdc* strain, which was created in this study. Complementation strains were generated by electroporation of pZL507 plasmids containing wild-type or mutant SdeA or SidC.

For *Legionella* infections, HEK293T cells were transfected with FCγRII and HA-Ub, EGFP-Rab1, 3xFlag-Rab1, 4xFlag-Rab33B or LULL1-HA for 24 hrs. Bacteria of indicated *Legionella* strains were opsonized with rabbit anti-legionella antibodies (1:500) at 37°C for 20 min before infection. The HEK293T cells were infected with post-exponential *L. pneumophila* strains at an MOI of 2 (for confocal imaging), or 20 (for Western blot) for the indicated amount of time.

### Immunoprecipitation and Western Blot.

For the enrichment of in vivo ubiquitinated substrates, Rab1, Rab33B, LULL1, or Stx17, transfected HEK293T cells were challenged with the Lp strains for the indicated amount of times and then the cells were lysed in ice-cold RIPA buffer (50 mM Tris pH 7.5, 150 mM NaCl, 1% Triton, 0.1% SDS, 0.5% deoxycholic acid) with protease inhibitor cocktail (Roche). Samples were sonicated and then centrifuged at 14,000 rpm for 20 min at 4 °C. The supernatant was incubated with 50% slurry of the EZview Red Anti-Flag M2 Affinity Gel Beads (Sigma, F2426), EZview Red Anti-HA Affinity Gel beads (Sigma, E6779) or GFP-nanobody conjugated resins for 2 hours for immunoprecipitation of 3xFlag-Rab1, 4xFlag-Rab33B, Flag-Stx17, HA-Rab33B, LULL1-HA, or EGFP-3xFlag-Rab1.

For co-immunoprecipitation, FCγRII, 3xFlag-Rab1, and HA-p62 transfected HEK293T cells were infected with specified Lp strains for 1 hour. Cells were then lysed in ice-cold lysis buffer (50 mM Tris pH 7.5, 150 mM NaCl, 1% Triton, 0.5% deoxycholic acid, 5mM EDTA, 5% glycerol) with protease inhibitor cocktail (Roche) for 30 min. 5% of the supernatant was used as input and the rest was mixed with anti-Flag beads for 2 hours for immunoprecipitation of 3xFlag-Rab1.

All Western blots were performed according to standard procedure. Briefly, samples were boiled and separated by gel electrophoresis with 12% or 15% SDS-PAGE gels and transferred to a PVDF (GE HealthCare) or nitrocellulose (MilliporeSigma) membrane. Membranes were incubated for 1 hour in 5% milk (Carnation) in TBST. Milk was washed and membranes were incubated overnight with indicated primary antibodies. Secondary antibodies used were Alexa Fluor donkey anti-mouse 680 (Invitrogen) or donkey anti-rabbit RDye 800CW (LI-COR) at 1:10,000 dilutions.

### Antibodies, immunostaining, and fluorescent microscopy.

Anti-HA (Sigma-Aldrich), anti-Flag (Proteintech), and anti-Ub (BioLegend) antibodies were purchased commercially. Anti-*L. pneumophila* antibodies were described previously ^45^. *Legionella* bacterium staining was performed as previously described ^45^. Infected HEK293T cells expressing FCγRII and HA-Ub, EGFP-Rab1, LULL1-HA, EGFP-sec61β, or mCherry-p62 were fixed using 4% PFA. Extracellular bacteria were incubated with rabbit anti-L. *pneumophila* primary antibody and then with Alexa Fluor donkey anti-rabbit 647 nm secondary antibody. The cells were then permeabilized with 0.1% triton and incubated with rabbit anti-L. *pneumophila* primary antibody, and then stained with Alexa Fluor donkey anti-rabbit 568 nm or 488 nm secondary antibody. HA-Ub or LULL1-HA was stained using a mouse-anti-HA primary antibody and donkey anti-mouse 488 nm secondary antibody.

Images were captured on a spinning disk confocal microscope (Intelligent Imaging Innovations, Denver, CO) with a spinning disk confocal unit (Yokogawa CSU-X1), a fiber-optic laser light source, an inverted microscope (Leica DMI6000B), a 100× 1.47 NA objective lens, and a Hamamatsu ORCA Flash 4.0 v2+ sCMOS camera. Images were captured using SlideBook 6.0 software and analyzed using ImageJ software.

### Mass spectrometry sample preparation.

Ubiquitinated EGFP-3xFlag-Rab1 or 4xFlag-Rab33B from wild-type Lp strain infected HEK293T cells was purified using anti-GFP-nanobody conjugated beads. 4xFlag-Rab33B in vitro ubiquitination reactions were performed as described above. For elution samples, EGFP-3x-Flag-Rab1 or 4x-Flag-Rab33B was eluted by incubating the resin (GFP-nanobeads or Flag-beads) with elution buffer containing 1% SDS in 100 mM Tris pH 8.0 at 65 °C for 15 min. For DUB cleavage samples, ubiquitinated EGFP-3xFlag-Rab1 or 4x-Flag-Rab33B samples were treated with 1 μM of SdeA DUB as described above to yield predominantly mono-Ub species. Eluted or DUB-treated samples were reduced with 10 mM DTT and alkylated with 25 mM iodoacetamide. Samples were then precipitated in PPT (0.1% glacial acetic acid, 49.9% ethanol, and 50% acetone) on ice for one hour. Proteins were pelleted and resolubilized in 8M Urea in 50 mM Tris pH 8.0. Samples were then digested with Pierce trypsin protease MS-grade (Thermo Scientific) at 37 °C overnight. Trypsinized samples were acidified with the addition of trifluoroacetic acid and formic acid, bound to a C18 column (Waters), and washed with 0.1% acetic acid. Peptides were eluted in 80% acetonitrile and 0.1% acetic acid and dried in a speed-vac. Samples were resuspended in 0.1 picomol/μL of angiotensin in 0.1% trifluoroacetic acid.

### Mass spectrometry and data analysis.

Peptides were analyzed using an EASY-nLC 1200 System (catalog no. LC140; Thermo Scientific) equipped with an in-house 3 μm C18 resin- (Michrom BioResources) packed capillary column (125 μm × 30 cm) coupled to an Orbitrap Fusion Lumos Tribrid Mass Spectrometer (catalog no. IQLAAEGAAPFADBMBHQ; Thermo Scientific). MS1 precursors were detected at *m*/*z* = 375–1500 and resolution = 240,000. The AGC target and maximum injection time were set at STANDARD and 50 ms. Precursor ions with a charge of 2+ to 7+ were isolated with an isolation window of 1.2 for MS2 analysis. Here, ions were isolated in the quadrupole and collected to standard AGC target and fragmented by high energy dissociation with a collision energy of 32%. Spectra were recorded using Thermo Xcalibur Software v.4.4 (catalog no. OPTON-30965; Thermo Scientific) and Tune application v.3.0 (Thermo Scientific).

Raw files were searched using Proteome Discoverer Software 2.3 (Thermo Scientific). The protein sequence database was the UniProt human database. The peptide identification and quantification pipeline relied on tools from the trans-proteomic pipeline (TPP). PhosphoRibose of arginine (+212.009 Da), and GG motif of lysine (+114.043 Da). Cysteine carbamidomethyl (+57.021 Da) was set as a static modification. Search parameters specified precursor mass and fragment mass tolerance of 15 ppm. Spectra were searched with the SEQUEST HT or Comet (v. 2019.01.1) search engine, and validated with Percolator or PeptideProphet algorithm. All results were filtered using the following parameters: the minimum probability of 0.9, minimum peptide length of 7 amino acid residues, accurate mass binning, and restriction to +2, +3, and +4 ion charge states. Phosphoribosylated peptides were then evaluated by PTMProphet to obtain a localization score for the modification ^72^.

## Extended Data
